# A Case of Uterine Pregnancy: Gastrotomy

**Published:** 1887-12

**Authors:** Wm. M. Cunningham

**Affiliations:** Bastrop, Texas


DANIEL'S

### TEXAS MEDICAL JOURNAL.

PUBLISHED MONTHLY.—SUBSCRIPTION $2.00 A YEAR.

Vol. 3. AUSTIN, DECEMBER, 1887. No. .

### ORIGINAL ARTICLES.

CONTRIBUTED EXCLUSIVELY TO THIS JOURNAL.

*The Articles in this Department are accepted and published with the understandin that we are not responsible for, nor do we indorse the views and opinions of the writer by so doing.*

### A CASE OF EXTRA UTERINE PREGNANCY AND GASTROTOMY.

*Reported by Wm. M. Cunningham, M. D., of Bastrop, Texas.*

Read before the Austin District Medical Society, and published by vote of the Publishing Committee.*

THE subject of this report, Mrs. Elizabeth W———, was an American lady, of the highest standing, in the good society of beloved old Bastrop.

I was called to see her for the first time, on the the 14th of last July, without an intimation as to the nature of her trouble. I found that her abdomen was very much distended, so much so, that her respiration was materially interfered with, and the heart action was very quick. Her temperature was 100° Fahrenheit, and her pulse 120 beats per minute. By palpation, I discovered, that the abdomen contained a fluid; and by pressure over the right inguinal region, I could feel a hard tumor, which was quite movable.

*) By vote of the Austin District Medical Society, *Daniel's Texas Medical Journal* was unanimously selected as the OFFICIAL ORGAN of the Society.

She said, she had known of a tumor about the size of a cocoanut existing there for a number of years—she did not know how long; but thought, it began about twenty years ago, during, or about the time of her monopause. She had no trouble, as she thought, during this change of life, and thought this tumor was due to the cessation of the menstrual flow. She said that she had been married twice; was now a widow; was the mother of nine children at eight births; and if she should live until the 24th day of January, 1888, she would be sixty-nine years of age. She had no difficulty in any of her labors; and her youngest child was born in 1858. Her health had always been good until about two years ago. She had not experienced any discomfort from the tumor until then. She would have to move it sometimes with her hand into a more comfortable position. Her appetite began to fail, and she felt a loss of energy and power of life. She could not bear cold weather, and could hardly be warmed, when she became cold. She had to be warmly clad even in summer. She felt tired, weak and much debilitated. She noticed, too, that the tumor was increasing in size. She had never experienced these conditions and changes before. With this history, and the conditions, I found on my first visit, I made a diagnosis at once, of compound ovarian cystic tumor of the right ovary.

On account of her feebleness, and great prostration, I decided that the treatment could only be palliative and symptomatic. In order to relieve the pressure, the distressed respiration, and quickened circulation, I decided to tap the cyst at once, and let out the great accumulation of fluid. This was repulsive to the family, and also to my patient; and it was delayed until July 25th. When, after giving her a dose of digitalis and morphine, I had her placed on her right side, near the edge of the bed, and three long strips of strong cloth placed around the abdomen, so that my assistants could produce pressure by pulling on the ends of these strips, from her back. I introduced a large trocar and canula in the median line half way between the umbilicus and symphysis pubis, removing the trocar and leaving the canula in position, and drew off three gallons of serous fluid, containing some albumen and a very little blood.

There was no syncope attending or following the operation; which is much less likely to occur in the horizontal than in the upright position. She felt relieved at once, and rejoiced that she could once more, take in a deep inspiration. I could now feel a long irregularly shaped tumor to the right of the median line, which was moveable in any direction, to every possible part of the abdominal cavity. This was conclusive that it had no parietal attachment. Introducing the sound into the uterus, I found its depth was about two and a half inches, and the fundus could be moved from right to left, without moving the tumor; hence it had no attachment to the uterus, and probably none to the bladder. My diagnosis now was that there truly existed a compound cystic tumor, with a pedicle attached to the right ovary, and it was likely that it had no other attachment; and if this be true, it could easily be removed by the operation of gastrotomy. But I decided, that it was too serious an operation for my patient, in her present condition, who was now much debilitated. She had no appetite, her pulse was very quick, she was restless, and nervous, and old. After adhesive plaster had been placed over the wound of the trocar, a supportive bandage was now placed around her abdomen, and she allowed to rest.

A tonic of iron, quinine, and strychnine, was prescribed to be given her three times daily; and directions were given that such nourishment as eggs, milk, broths, etc., should be given every four or five hours, during the day. She was given twenty grains of bromide potassium, fifteen minims of the tincture of digitalis, and one fourth grain of morphine, every night.

This acted beautifully, giving her rest and sleep. Her bowels, on account of the pressure of the tumor, scarcely ever moved, was made to act every other day, by either an enema or a dose of the sulphate of magnesia.

Under this course of treatment, she improved beyond my expectation. She soon regained her appetite; her pulse reduced to eighty; her temperature became normal; her bowels would occasionally act without being induced, and she was able to go about the house. I began to think it possible, that she might stand the operation of having the tumor removed, as the indications were, that its attachment was single, and to the righr ovary. Consequently, I submitted it to her, and to her family, as the only way of getting rid of the tumor, and as offering the only hope of her ultimate recovery; that, if it were left alone, it would soon destroy her life, as it had begun to develop itself, and to interfere with the functions of the abdominal organs. I told them of the seriousness of the operation. From this fact a surgeon would be justifiable in opening the abdomen, and removing the tumor.

The history of such tumors is, that when they begin to develop, and enlarge, to such an extent, as to interfere with the proper functions of the abdominal organs, they then begin to undermine and break down nutrition, and if left alone, they have but one ending, and that ending is destruction of life. There is no exception.

This was repugnant to my patient, and to her family; and I hope, it is allowed me to say, that it was repugnant also to her physician. It is a very serious operation, and its forebodings are sadness, and a great sense of responsibility. He submitted the operation, and his willingness to do it, or have it done, because he felt that it was his duty. In a few days after this last interview, my patient and her family had considered this operation, and decided, that if it offered the only chance for her recovery, she and her family wanted it done.

I immediately asked for consultation. Drs. W. G. V. Hickman, of Smithville, C. S. Tatum, of Weimar, and Samuel Cunningham, my brother, of Elgin, were each separately called, and they each, and every one, agreed with me in the diagnosis, and that if she were left alone, without the operation, she would soon die, and that if the tumor were removed, she would have some chance to get well, and they advised the operation. Our decision was given to the family, and to my patient. We discussed the propriety of keeping her on a tonic course of treatment, until the weather became cooler; but decided, that delay in her case, was dangerous. We accordingly set some day in the following week for the operation.

But before the time came, she began to loose her appetite, the accumulation was taking place rapidly in the abdominal cavity, so much so, that on the eight day of August, I tapped her cyst again, using the same method and precaution, as in the first case, and drew off three gallons more of this serous fluid. She began to improve at once. The wound of the trocar, as in the first instance, healed by first intention. And although the solid portion of the tumor was still enlarging, and the accumulation of fluid rapidly taking place, she was soon thought to be in as good a condition to stand the operation as she would ever be. Accordingly on the 17th day of August, Drs. W. G. V. Hickman and Samuel Cunningham, having been called back, we decided to operate. Her bowels were moved the evening before with a dose of the sulphate of magnesia, and on the morning of the operation, with an enema. Her bladder being emptied, she was placed on the table, and anæsthesia was produced by the inhalation of sulphuric æther, until anæsthesia was almost complete, when it was discontinued, and Squibb's chloroform used during the operation.

Our instruments and hands having been disinfected, we made an incision, beginning about two inches below the umbilicus, extending about four and a half inches in the median line, towards the symphysis pubis, down to the peritoneum. We stopped until all hemorrhage had been checked. Then catching up the peritoneum, with a tenaculum at the lower angle of the incision, perforating it, we introduced a grooved director, and slit it up with a sharp bistoury to the upper angle of the incision.

We expected to find the walls of the cyst just beneath the peritoneum—but not so. The abdominal cavity contained this serous fluid. We placed a large flat sponge over the incision, turned the patient on her right side, and let all this fluid run out; there was about two gallons. After placing her on her back, we introduced our hand, and found, that the tumor had no parietal attachment; that it was not attached to the uterus, nor to either ovary. We raised it gently out of the abdominal cavity, and found that it was an abdominal pregnancy, in which the fœtus had become ossified. We found that almost surrounding the ossified fœtus, was a fleshy mass, and a thick, dense walled fluctuating cyst. The whole tumor had five attachments to the mesentery, the small bowels, and to the great omentum, two of which were very strong. It was largely supplied with blood vessels, from the mesenteric and omental arteries and veins. The blood vessels were unusually large, and their walls very thin. One artery, just before entering the tumor, had pouched out into an aneurism, about the size of an English walnut, and looked as if it were just ready to burst. We decided, as we had gone thus far, and fearing fatal hemorrhage at any moment, we would complete the operation. Accordingly, after catching up the attachments of the tumor, being careful not to catch up a nuckle of intestine with the ovariotomy forceps, on account of the collateral circulation, we placed two white silk ligatures on each of the attachments, one below, and the other above the forceps, and divided them between the ligatures. Thus we removed the tumor without much hemorrhage. We sponged out the abdominal cavity until we had cleaned it as well as we could.

Then we brought the wound together, using silver wire for the deep quilled sutures, and white silk for the superficial interrupted sutures. We were careful to see that the opposite walls of the peritoneum were brought in juxtaposition. After putting on adhesive plaster, a roller of carbolized surgeons lint and an abdominal bandage, we placed our patient on a more comfortable bed, and the operation was finished. A hypodermic of morphine was given, her face bathed with cold water, warmth applied to her feet, and the anæsthesia was soon gone. She soon rallied, and talked with her children.

By dissecting the tumor, we found that only the back of the body, the trunk, hips, shoulders, neck and occiput, could be recognized as a fœtal formation, and was of the consistency of bone. It was about five inches in length, and two inches in breadth. Cutting the fœtus from the soft portion of the tumor, we found that its face, arms, legs, and its whole front, had perished and was wanting. Attached to the right shoulder by a bony connection, was another ossified formation, resembling very much the back portion of another head, though this was not as perfect, as the head attached, to the body by the neck. The soft portion of the tumor into which the fœtus was partially buried, did not differ from the texture and consistency of a placental formation. When we cut into the cyst, about a quart and a half of semi-fluid blood escaped. The weight of the whole tumor was seven pounds.

We reported the nature of the tumor to the family. Whereupon, the oldest daughter told me, that her mother did think, she was pregnant about twenty-one years ago, but that her menopause came on shortly afterwards, or about that time, and she considered it all due to that. We thought best not to tell our patient that it was an abdominal pregnancy. Of course, our prognosis was very unfavorable, when we consider the attachments, and the kind of tumor we removed in this case. Had it been an ovarian cystic tumor with only one attachment to the ovary, the prognosis would have been more favorable. But there is no way by which a differential diagnosis can be made between a compound ovarian cystic tumor, and a long standing abdominal pregnancy, where the history of the case was so imperfect, without cutting down on the tumor.

The shock of the operation was considerable ; but not as much as we expected to see in an aged woman like her. She suffered a little from nausea, and vomiting, for twenty-four hours. Her temperature was a little below normal, 97° to 98° Fahrenheit, during the first sixteen hours, when it began to rise. On the second day, her temperature had become normal, and her pulse below a hundred. She was cheerful, and remarked, that if she had known, the operation was no more severe than the one she had passed through, she would have known that she could have stood it and lived. She took some nourishment during the day, consisting of milk and egg-nogg. Quinine and opium were given from the beginning of the after treatment, to prevent movement of the bowels, and as a prophylatic to inflammation. Icebags were applied to the abdominal wound for the same purpose, and to prevent too much heat in the parts. She rested well all night, and felt more cheerful on the third morning. She wanted a cup of coffee, which was given her. The same treatment of the previous day was continued, and she did well until about six o'clock in the evening. She had been sleeping from the effects of opium, and on waking, she asked for water, which was given her with a large spoon while laying on her back. She choked on the water, and in an effort at relief, turned herself quickly and violently on her side. She hurt herself in the turn. A constant nausea, and an effort at vomiting, continued to such an extent as to prevent her from speaking for an hour. A cold perspiration came out on her face and hands. Her pulse became small, rapid and unsteady. The symptoms were so alarming, that I feared fatal hemorrhage had taken place. I gave her a hypodermic of morphine, and she soon dropped off into a restless sleep ; digitalis was also given. These symptoms continued until morning, when she seemed much revived, but did not feel as well as the morning before. Her temperature was 101° Fahrenheit, and her pulse 108 per minute. There was some tympanites of the abdomen.

The abdominal wound was examined, and found healing nicely. She complained of a little tenderness on pressure over the abdomen. The application of cold, and the administration of opium were continued. Digitalis and quinine were also continued. But there was no abatement of temperature, nor did it rise any higher. She became a little more cheerful as the day passed off, until evening, about six o'clock, when the symptoms again grew alarming, but not so much as the night before. We watched her carefully all night. Her temperature did not rise, but her pulse ran up to a hundred and twenty, and was a little unsteady.

She passed through the night and was revived beyond my expectation. This was on the morning of the fifth day after the operation. Her temperature remained at 101° Fahrenheit during the day, and her pulse came down to ninety-six per minute, occasionally was a little more frequent. Her urine had to be drawn off with the catheter, as it had been every eight and twelve hours. She felt a desire, for the first time, since the operation, for her bowels to move. A large rubber tube was introduced above the splhincter-ani muscle. A good deal of gas escaped but no fecal matter, and she felt somewhat relieved. She took some nourishment with-a little relish ; but she was nervous and tremulous all the day. Late in the evening she became delirious. Her pulse ran up and was feeble, unsteady and irregular, and the symptoms were more alarming than ever.

I called the family together, and told them that there was no hope, that she would die.

By the use of stimulants, she lived through the night; but at morning dawn, she died from exhaustion and heart failure.

My theory in this case is, that pregnancy must have taken place many years ago, somewhere within the fimbriated extremity of the right fallopian tube, or within the ovary itself; and within the first three weeks of gestation, before the formation of the placenta, the embryo was expelled into the abdominal cavity, invested in its decidua. The placenta formed and made its attachments to the mesentery, the bowels and omentum. Gestation continued for four or five months, or longer, when the fœtus died. Being preserved from decay, it underwent a gradual change, and became of the consistency of bone, after many years. The amniotic fluid must have been absorbed, and the hard, bony fœtus became embedded in its own placenta, and acted as a foreign irritating substance; hence, this whole mass of fecundation, developed into a large tumor. By reason of its presence, its growth, and its pressure, the abdominal membranes poured out a serous fluid into the abdominal cavity—thus adding pressure to pressure, which greatly interfered with the action of the abdominal organs, and finally led to this operation.

It is true, that this lady was very old to have undergone so serious an operation, but it gave her the only chance for recovery. If it had been left alone, she would have soon died, as do all cases of abdominal and ovarian tumors, which develop in size and arrest the functions of the vital organs, when left alone, unrelieved by surgery. Surgeons do not seem to take into much consideration the age of a patient, when they contemplate the operation of ovariotomy, or gastrotomy. They prefer their patient to have passed that climax in life, in which menstruation has ceased. Statistics show that the operation is far more successful after the menopause, than during the age of menstruation.

In all cases of non-malignant ovarion abdominal tumors, which are movable, whose attachments are not extensive, and which have no attachment to the liver, if they have begun to develop in size, and to interfere with the proper functions of the abdominal organs, and if the constitution and vital powers of the patient are reasonably good, the operation of ovariotomy, or gastrotomy, for the removal of these tumors is perfectly justifiable at any age in life.

